# *Wolbachia* Infection Reduces Blood-Feeding Success in the Dengue Fever Mosquito, *Aedes aegypti*

**DOI:** 10.1371/journal.pntd.0000516

**Published:** 2009-09-15

**Authors:** Andrew P. Turley, Luciano A. Moreira, Scott L. O'Neill, Elizabeth A. McGraw

**Affiliations:** 1 School of Biological Sciences, The University of Queensland, St. Lucia, Queensland, Australia; 2 Rene Rachou Research Institute- FIOCRUZ, Belo Horizonte, Brazil; National Institute of Allergy and Infectious Diseases, United States of America

## Abstract

**Background:**

The mosquito *Aedes aegypti* was recently transinfected with a life-shortening strain of the endosymbiont *Wolbachia pipientis* (*w*MelPop) as the first step in developing a biocontrol strategy for dengue virus transmission. In addition to life-shortening, the *w*MelPop-infected mosquitoes also exhibit increased daytime activity and metabolic rates. Here we sought to quantify the blood-feeding behaviour of *Wolbachia*-infected females as an indicator of any virulence or energetic drain associated with *Wolbachia* infection.

**Methodology/Principal Findings:**

In a series of blood-feeding trials in response to humans, we have shown that *Wolbachia*-infected mosquitoes do not differ in their response time to humans, but that as they age they obtain fewer and smaller blood meals than *Wolbachia*-uninfected controls. Lastly, we observed a behavioural characteristic in the *Wolbachia* infected mosquitoes best described as a “bendy” proboscis that may explain the decreased biting success.

**Conclusions/Significance:**

Taken together the evidence suggests that *w*MelPop infection may be causing tissue damage in a manner that intensifies with mosquito age and that leads to reduced blood-feeding success. These behavioural changes require further investigation with respect to a possible physiological mechanism and their role in vectorial capacity of the insect. The selective decrease of feeding success in older mosquitoes may act synergistically with other *Wolbachia*-associated traits including life-shortening and viral protection in biocontrol strategies.

## Author Summary

The primary mosquito vector of dengue virus, *Aedes aegypti*, has recently been artificially infected with a symbiotic bacterium called *Wolbachia pipientis*. This bacterium occurs naturally inside the cells of ∼66% of insect species. The *Wolbachia* used to infect *A. aegypti* shortens the insect's lifespan. Because only old mosquitoes are capable of transmitting dengue virus, the *Wolbachia* infection could theoretically reduce dengue virus transmission if infected mosquitoes were released into the wild. Here we have examined the effects of this *Wolbachia* infection on the mosquito's ability to obtain blood meals from human hosts. Blood is required for females to produce eggs, and so successful completion of this behaviour is necessary if *Wolbachia*-infected mosquitoes are to be competitive in the wild. Blood feeding on humans is also the time when viruses like dengue are transmitted, so changes in this behaviour can have consequences for the transmission rate of viruses. We show that *Wolbachia*-infected mosquitoes are less able to obtain blood meals, but only in old age. The reduced feeding success may be explained by a defect in the insect's proboscis. The finding is exciting as it may allow young mosquitoes to breed as normal but help reduce the lifespan and success of old mosquitoes, which are the primary transmitters of virus.

## Introduction

A number of the major mosquito vectors of disease, including the various anopheline species that transmit malaria and *Aedes aegypti* that transmits dengue and other arboviruses, do not naturally carry the common insect endosymbiont *Wolbachia pipientis*. Recently, *A. aegypti* was artificially transinfected in the laboratory with a virulent, life-shortening strain of *Wolbachia*, *w*MelPop, that is native to *Drosophila melanogaster* [1]. In *D. melanogaster* the infection is thought to shorten the insect's lifespan by over-replicating and causing rupture of host cells [2]. *A. aegypti* were transinfected with this *Wolbachia* strain with the aim of developing a novel biocontrol strategy based on reducing mosquito lifespan in the field. Such a shift in mosquito population age structure could theoretically reduce or even eliminate dengue virus transmission given that only old mosquitoes transmit dengue [3],[4].

The *w*MelPop infection demonstrated a range of effects on its non-natural host including the predicted and sought after phenotypes of cytoplasmic incompatibility and life-shortening [1]. The former phenotype is common to most insect-*Wolbachia* associations and is predicted to serve as a driving mechanism for spread of *Wolbachia* infections in the field [5]. The latter is a trait that appears to be uniquely associated with the *w*MelPop strain [2]. The *w*MelPop-infected mosquitoes also exhibit increased daytime locomotor activity and metabolic rates [6]. These two *Wolbachia*-associated effects suggest physiological differences between infected and uninfected insects that could affect complex behaviour like mate seeking and foraging. In blood-feeding insects, performance in these complex behaviours can dramatically affect the fitness of individual mosquitoes and the frequency of disease transmission [7],[8]. *Wolbachia*-associated changes in insect activity have also been previously reported for both a *Drosophila* parasitoid [9] and two *Drosophila* species [10].

Infection-induced increases in hunger and foraging rates have been demonstrated for other insect systems including microsporidia infected honeybees [11] and parasitoid infected aphids [12]. Increased hunger is one possible explanation for the activity of *Wolbachia*-infected *A. aegypti*, as a sugar water source was present in the chambers where mosquito activity was measured [6]. *Wolbachia* could either be causing increased hunger indirectly, by forcing the host to expend energy by mounting an immune response [13] or directly, by accessing host resources for its own nutrition [11]. To date there is no documented evidence of *Wolbachia* inducing an immune response in insect hosts [14], although the effects of *w*MelPop infection have been less well explored. Sequencing of the *Wolbachia* genome, however, has revealed clear points where *Wolbachia* must rely on hosts due to missing or incomplete biochemical pathways, particularly with respect to amino acid biosynthesis [15],[16]. The presence of any such *Wolbachia*-associated drain on host resources may historically have been missed in fitness assays performed in nutrient rich, ideal laboratory conditions [17].

Changes in insect activity due to *Wolbachia* could, however, simply be a by-product of the infection process. *Drosophila* with systemic bacterial infections, for example, commonly show altered circadian rhythms and sleep patterns [18]. In the *Wolbachia* system, the use of the term “virulence” has traditionally been reserved for describing the life-shortening phenotype of *w*MelPop [19]. Our definition of virulence may need to expand to include these other behavioural and physiological consequences of infection associated with a broad range of strains [9],[10]. As *Wolbachia* infections are highly dispersed throughout the bodies of insects [2],[20],[21] they have the potential to alter the function of a wide range of tissues.

Here we sought to examine the effects of *w*MelPop infection on *A. aegypti* blood-feeding behaviour. In both small population and individual trials, we assessed mosquito response time to human hosts, frequency of successful blood feeding, and blood-meal weights. These characteristics were compared for *w*MelPop infected and uninfected control mosquitoes at a range of ages. The aim was to test for evidence of *Wolbachia*-associated energetic drain, as revealed by increased foraging behaviour, or *w*MelPop-associated virulence effects on mosquito feeding. An examination of both old and young mosquitoes was critical to capture any potential increase in virulence with age as predicted by the *D. melanogaster*-*w*MelPop association [2].

## Materials and Methods

### Ethics statement

This study was conducted according to the principles expressed in the Declaration of Helsinki. The study was approved by the Medical Research Ethics Committee at the University of Queensland (Project #2007001379). All patients provided written informed consent for the collection of samples and subsequent analysis.

### Mosquito rearing

For all experiments two laboratory lines of *A. aegypti* were used, the PGYP1 line, previously generated by transinfection with *w*MelPop and PGYP1.tet, its *Wolbachia* cured control line [1]. Mosquitoes were reared at 26±2°C, RH 75% with 12 h∶12 h light/dark cycle. Larvae were fed 0.1 mg/larvae of TetraMin Tropical Tablets once per day. Females were separated from males at the pupal stage and placed into 300 mm^3^ cages for emergence at a density of 400 individuals per cage. The females were fed 10% sucrose solution *ad libitum* until the day before feeding trials.

### Confirmation of infection status

Mosquito lines were screened to confirm presence (PGYP1) or absence (PGYP1.tet) of infection every two generations using a PCR based assay. Five days after eclosion, DNA was extracted from 10 females using DNeasy spin columns (QIAGEN, Australia), following the manufacture's protocol. PCR was then carried out using primers for the IS5 transposable element present in *Wolbachia* [22]. Reaction conditions were as follows: 0.01–0.09 µg of each DNA sample, 2 µl of 10× Buffer, 0.5 µl 1 mM dNTPs, 0.5 µl of 20 µM IS5 primers, 0.15 µl Taq DNA polymerase and water up to 20 µl. Samples were denatured for three minutes at 94°C then cycled 34 times for 30 seconds at 94°C, 30 seconds at 55°C and one minute at 72°C. This cycle was followed by a final 10-minute extension at 72°C. Presence of the expected size product was then confirmed by agarose gel electrophoresis.

### Preparation for feeding trials

Experiments were conducted with five, 26 and 35-day-old adult mosquitoes. Behaviours were measured in either small populations (proportion of population fed and number of attempted bites) or for single mosquitoes (response time to human, blood-meal weight and length of attempted bites) depending on feasibility. The afternoon prior to each trial the required number of mosquitoes were removed from their rearing cages and stored in mesh-covered holding buckets at a density of five mosquitoes per bucket. At the same time an additional population of five mosquitoes were set aside to replace any mosquitoes that died during the starvation period. Mosquitoes were starved of sucrose but given access to water for ∼16 hours until trials began the next morning. Prior to each trial, mosquitoes were transferred from holding buckets into a 645 cm^3^ cage and allowed to acclimatise for 5 minutes. All human volunteers cleaned both of their forearms with 70% isopropyl alcohol wipes, rinsed their forearms with distilled water and dried them with paper towel, and placed latex gloves on both their hands before feeding.

### Population trials

All population trials were carried out in two cages placed next to one another. One cage contained five PGYP1 mosquitoes and the adjacent cage contained five PGYP1.tet mosquitoes. The position (left or right) of the two lines was alternated throughout the experiment. Volunteers inserted an arm into each of the two cages and rested their hands on buckets placed within each cage. Both the volunteer and an external assistant monitored the number of attempted bites each mosquito made on the volunteer's forearm. An attempted bite was recorded when a mosquito landed and actively attempted to probe the volunteer's skin at a location. A single mosquito could attempt to probe multiple times at a single location, but if a mosquito moved to a new position and attempted to probe again, then this new location was recorded as another attempted bite. Mosquitoes in both cages were monitored for 15 minutes before the volunteer shook their arms and withdrew both arms from the cages. Mosquito abdomens were examined for presence of a blood meal and the proportion of the population that imbibed a blood meal was recorded. This experiment was replicated with six volunteers (3 female, 3 male) ×4 replicate trials for each of the three adult mosquito age classes.

### Individual trials

A single mosquito from each line was separately aspirated into a pre-weighed 1.5 ml microcentrifuge tube and weighed on an analytical balance. Each mosquito was then released simultaneously into the adjacent 645 mm^3^ cages. Alternation of cage position, mosquito settlement time and trial length were as per population trials. The volunteer inserted their arms into the cage and the times at which mosquito's made their first attempted bite (host-seeking time) and the length of each attempted bite were recorded by the volunteer into a voice recorder. After the trial, mosquitoes were transferred back into the tubes they were originally weighed in and the tubes were re-weighed. The weight of the blood-meal imbibed by each mosquito was then calculated. The volunteer (male) hosted four groups of 10 mosquitoes from each of the three age classes.

### Statistical analysis

All analysis was conducted using STATISTICA v8 (StatSoft, Inc). The variables, host-seeking time, length of attempted bites and blood-meal weight were normally distributed. The number of attempted bites was square root transformed to achieve normality. The role of infection and age on these variables was examined using general linear mixed models. The role of human volunteer was not examined as there were only 6 replicate individuals and they were internally controlled. When infection status was significant, t-tests were then used to further identify specific differences between infected and uninfected lines within each of the three age classes. The proportion of mosquitoes obtaining a blood meal did not respond to transformation and so non-parametric Mann Whitney U-tests tests were employed instead of linear models to examine differences between infected and uninfected mosquitoes for all three ages.

## Results

### Host seeking

**Figure 1:**
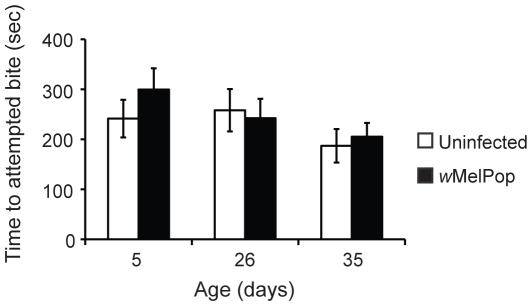
Time until first attempted bite. Bars represent means±sem from individual trials. No significant differences were observed between infected and uninfected mosquitoes for any of the ages. N = 31–33 per treatment.

If the *Wolbachia*-infected mosquitoes were hungrier than uninfected counterparts they might be expected to exhibit a more rapid response to the human forearm. Over the short distance in the laboratory cage environment, infected mosquitoes did not differ (*F* = 0.10, df = 1, *P* = 0.77) in the time it took them to land on the human volunteer and initiate an “attempted bite” (Fig. 1). Age of the mosquitoes was also not a significant determinant of length of time to first “attempted bite” (*F* = 0.99, df = 2, *P* = 0.43). These data suggest that *w*MelPop does not alter mosquito capacity to sense and respond to human hosts in the laboratory-based assay.

### “Attempted biting”

**Figure 2:**
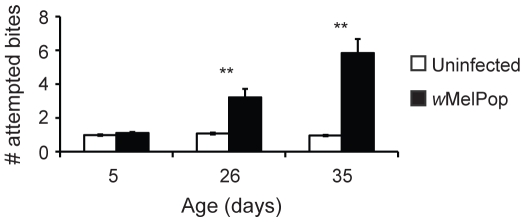
Number of attempted bites. Bars represent means±sem from population trials. *P<0.05, **P<0.001 by t-test. N = 117–120 per treatment.

The number of “attempted bites” made by infected mosquitoes was also examined as a possible indicator of hunger. As per our methods, an attempted bite encompassed both probing and attempted probing in a particular region on the arm. Given the cage sizes and numbers of mosquitoes involved we could not visually differentiate between a probing event that broke the skin and one that did not. Infection status (*F* = 13.37, df = 1, *P* = 0.014), age of mosquitoes (*F* = 5.72, df = 2, *P* = 0.021), and the interaction between age and infection status (*F* = 5.76, df = 2, *P* = 0.021) were significant determinants in the number of attempted bites made. In particular, *Wolbachia*-infected mosquitoes at 26 (*t* = −3.70, df = 238, *P*<0.001) and 35 days of age (*t* = −5.35, df = 235, *P*<0.001) attempted to bite more than their uninfected counterparts (Fig. 2). This was not the case for five-day-old mosquitoes (*t* = −1.12, df = 236, *P* = 0.26). The significant interaction between infection status and age as reported above is seen in the increase in biting attempts by infected mosquitoes in the older age classes (Fig. 2). For example, if we directly compare infected 26-day-old versus 35-day-old mosquitoes we see an increase (*t* = −2.70, df = 235, *P* = 0.0073) in the mean number of attempted bites while this is not the case for uninfected mosquitoes (*t* = 1.72, df = 238, *P* = 0.085). Lastly, we also measured the length of time each mosquito spent on an attempted bite (data not shown), which was not influenced by infection status (*F* = 0.75, df = 1, *P* = 0.45) or age (*F* = 1.68, df = 2, *P* = 0.26) of the mosquitoes. These data suggest that as *Wolbachia*-infected mosquitoes age they are exhibiting a greater number of attempted bites than uninfected mosquitoes, but are not spending more time on any one attempt.

### Blood meal acquisition

**Figure 3:**
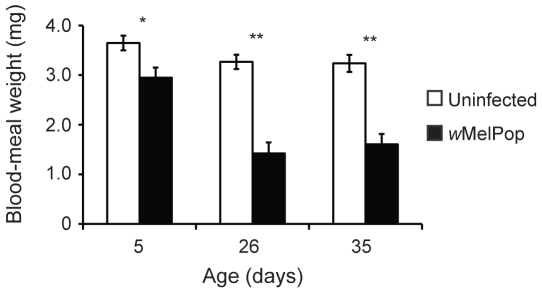
Weight of imbibed blood meal. Bars represent means±sem from individual trials. *P<0.05, **P<0.001 by t-test. N = 31–33 per treatment.

**Figure 4:**
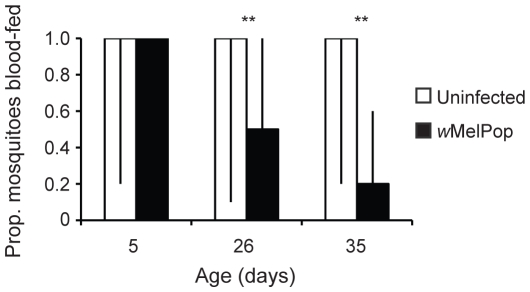
Proportion of the population that imbibed a blood meal. Bars represent medians ±25% and 75% quartile values from population trials. **P<0.001 by Mann Whitney-U test. N = 117–120 per treatment.

Blood-meal weight (Fig. 3) was examined as a measure of feeding success in the infected mosquitoes. Linear models revealed that blood-meal weight could be partially explained by the infection status (*F* = 87.07, df = 1, *P*<0.001) and age of mosquito (*F* = 16.87, df = 2, *P*<0.001). There was also a significant interaction between age and infection status (*F* = 5.59, df = 2, *P* = 0.004). The blood-meal weight of *w*MelPop-infected mosquitoes was smaller than uninfected mosquitoes for all ages examined, (5 d, *t* = −2.80, df = 67, *P* = 0.007; 26 d, *t* = −7.15, df = 67, *P*<0.001; 35 d, *t* = −6.09, df = 66, *P*<0.001) with the differential increasing with age (Fig. 3). If infected mosquitoes were on average smaller than their uninfected counterparts, then smaller blood-meal weights would also be expected. A comparison of average weights of the infected and uninfected mosquitoes' pre-blood meal indicated there were no size differences between the lines, PGYP1 and PGYP1.tet (df = 204, *t* = 1.57, *P* = 0.11). The median proportion of mosquitoes that imbibed a blood meal (Fig. 4) was also reduced for infected 26 (*Z* = 4.10, *P*<0.001) and 35-day-old (*Z* = 5.39, *P*<0.001), but not 5-day-old (*Z* = 0.83, *P* = 0.74) mosquitoes relative to uninfected. These data indicate that as *Wolbachia* infected mosquitoes age, an increasing proportion of the population fails to successfully obtain a blood meal and that when they do feed the meals are smaller.

### Behavioural observations

**Figure 5:**
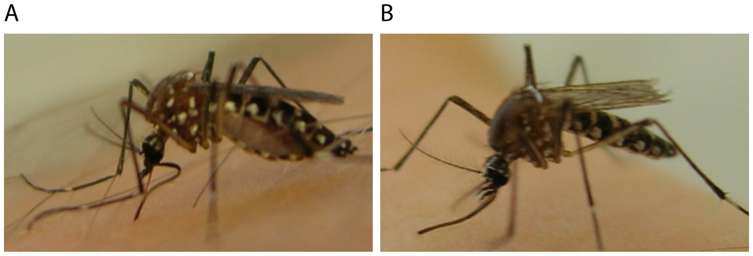
Abnormal biting behaviour in old *w*MelPop infected mosquitoes. Successful (A) and unsuccessful (B) probe by *A. aegypti* mosquitoes. In A. the proboscis of this *w*MelPop-infected mosquito is bending as it thrusts its head towards the volunteer's skin.

Normally during biting a mosquito may probe unsuccessfully, but will ultimately insert its stylet into a host (Fig. 5A). In this study, infected mosquitoes were observed in which the proboscis repeatedly bent as the mosquito pushed its head towards the skin while probing (Fig. 5B, Video S1). This phenotype was present only in *w*MelPop-infected mosquitoes, typically of 35 days of age.

## Discussion

We have identified changes in *A. aegypti* blood-feeding behaviour in response to infection with the virulent strain of *Wolbachia*, *w*MelPop. As infected female mosquitoes age they experience increasing difficulty in successfully obtaining a blood meal and the size of the blood meal becomes smaller. With increasing failure to feed, we also see an increase in the number of “attempted bites” made by the infected mosquitoes. Five-day-old females do not show decreased success in feeding, but do show decreased blood-meal size relative to uninfected mosquitoes. Infected mosquitoes, despite all of these differences, do not exhibit a change in responsiveness to human hosts. Lastly, we observed a behavioural trait we term “bendy proboscis” in *w*MelPop-infected mosquitoes that could potentially explain failure of individuals to successfully obtain blood meals.

There are two outstanding questions generated by this study. The first is that while we quantified the numbers of “attempted bites” we were not able to differentiate the proportion of individuals whose stylet successfully pierced the skin. An understanding of this trait is critical as it directly relates to the insect's capacity to vector pathogens. A subsequent study by our laboratory is currently exploring how *w*MelPop infection affects the frequency of successful skin piercing as the mosquito ages. Second, given the discovery of the “bendy proboscis” phenotype late in the study in older individuals, its occurrence relative to age and infection status was not quantified. We are currently developing a quantitative relationship between the bendy trait, infection status, age, and lack of feeding success to test our hypothesis regarding its role in failed feeding.

Our original aim was to use response time to humans, blood-meal size and feeding success to test for underlying differences in hunger between infected and uninfected mosquitoes. Feeding increases in response to parasites have been documented previously. In many of these cases, where the parasite is transmitted via the blood meal, increased feeding tends to be explained as a parasite manipulation of the host for improving its own transmission [23],[24],[25]. As *Wolbachia* is maternally inherited its transmission is not confounded with blood feeding. Models like that of microsporidia in honey bees [11] and parasitoids of aphids [12] are more appropriate for comparison and do show evidence of direct effects of parasites on host hunger. The heightened daytime activity of *w*MelPop infected *A. aegypti*, revealed by videography [6], could have been explained by more frequent trips of the mosquito to the sugar water source present in the recording arena. Further studies in our laboratory also indicate that infected individuals on average take smaller sugar-water meals than uninfected mosquitoes (unpublished data). Without an equal ability to obtain similar sized meals or equal probability of success with respect to feeding, basal “hunger” of infected and uninfected mosquitoes could not easily be compared.

The reduced blood-feeding success and evidence of the “bendy proboscis” defect in *w*MelPop-infected mosquitoes, point toward a model of infection induced virulence. Disruption of host activities or damage at the level of the tissue or cell could lead to consequences for host physiological function and hence complex behaviours like feeding. In particular, host neuropeptides that regulate feeding behaviour are an appealing target for mechanistic exploration [26]. The pattern seen here of worsening effect with increasing age parallels the predicted virulence in the *w*MelPop:*D. melanogaster* association, where as bacterial densities rise, host cells lyse in association with host mortality. Indeed there are few published studies examining *w*MelPop properties such as growth kinetics and induced damage at the level of individual tissues. Even in *D. melanogaster*, 20 years after its initial report [2], the virulence properties of *w*MelPop and the nature of the host response may have evolved [19]. With *w*MelPop now stably infecting *D. melanogaster* [2], *D. simulans* [19], and *A. aegypti* [1] and present in the somatic tissue of *Anopheles gambiae* [27], these associations are ripe for comparative study at the tissue level. The suite of effects identified here in *A. aegypti* render the nervous tissue, musculature, proboscis and digestive tract of particular interest.

This work highlights the growing diversity of *Wolbachia*-induced host somatic phenotypes, and offers the first report of effects for a blood-feeding insect. The nature of the behavioural changes suggests they are the result of infection-induced virulence. A determination of the physiological basis of the virulence effects requires further examination at the cellular or tissue level. The changes in feeding behaviour documented here may further improve *w*MelPop's ability to limit transmission of human pathogens in association with the traits of life-shortening [1] and the more recently documented dengue viral protection (Moreira, unpublished.) in *A. aegypti*. By limiting the blood-feeding success of old mosquitoes we could further reduce the period where the mosquito could function as a disease vector. As the blood-feeding effect is present only in older mosquitoes the reproductive capacity of young mosquitoes should theoretically be preserved. The balance of the onset of feeding difficulties versus the primary window of mosquito reproductive output will in part determine the virulence of the *w*MelPop on the host and possibly affect its long-term stability in an evolving population.

## Supporting Information

Video S1 Bending proboscis movie. Video file of mosquito with bending proboscis, infected with wMelPop, aged 35 days.
(8.99 MB MPG)
